# High-molecular-weight adiponectin and anthropometric variables among elementary schoolchildren: a population-based cross-sectional study in Japan

**DOI:** 10.1186/1471-2431-12-139

**Published:** 2012-09-01

**Authors:** Hirotaka Ochiai, Takako Shirasawa, Rimei Nishimura, Aya Morimoto, Tadahiro Ohtsu, Hiromi Hoshino, Naoko Tajima, Akatsuki Kokaze

**Affiliations:** 1 Department of Public Health, Showa University School of Medicine, 1-5-8 Hatanodai, Shinagawa-ku 142-8555, Tokyo, Japan; 2 Division of Diabetes, Metabolism and Endocrinology, Department of Internal Medicine, Jikei University School of Medicine, 3-25-8 Nishi-Shinbashi, Minato-ku 105-8461, Tokyo, Japan; 3 Jikei University School of Medicine, 3-25-8 Nishi-Shinbashi, Minato-ku 105-8461, Tokyo, Japan

**Keywords:** High-molecular-weight adiponectin, Anthropometric variable, Obesity, Waist-to-height ratio, Children

## Abstract

**Background:**

Studies about the relationship between high-molecular-weight adiponectin (HMW-adn) and anthropometric variables among population-based elementary schoolchildren have been too limited, especially in Japan, where blood collection is not usually performed in the annual health examination at elementary schools. The objective of the present study was to investigate the relationship between HMW-adn and anthropometric variables (body mass index [BMI], percent body fat [%BF], waist circumference [WC], and waist-to-height ratio [WHtR]) among population-based elementary schoolchildren in Japan.

**Methods:**

Subjects comprised all fourth-grade schoolchildren (9 or 10 years of age) in the town of Ina, Saitama Prefecture, Japan during 2005–2008 (N = 1675). After excluding 21 subjects because of refusal to participate or incomplete data, data from a total of 1654 subjects (846 boys and 808 girls) were analyzed. The height, weight, %BF, and WC of each subject were measured, while blood samples were drawn from the subjects to measure adiponectin levels (HMW-adn and total adiponectin). Childhood obesity was determined according to the age- and sex-specific cut-off points proposed by the International Obesity Task Force. Spearman’s correlation coefficients between adiponectin levels and anthropometric variables were calculated for each sex.

**Results:**

The anthropometric variables were negatively correlated with HMW-adn in both boys and girls. Correlation coefficients of HMW-adn with anthropometric variables in the obesity group were consistently higher than those in the non-obesity group among both boys and girls. In addition, only WHtR was significantly correlated with HMW-adn regardless of sex and physique (obesity or non-obesity); the correlation coefficient was -0.386 among boys and -0.543 among girls in the obesity group, while it was -0.124 among boys and -0.081 among girls in the non-obesity group.

**Conclusions:**

HMW-adn was negatively correlated with anthropometric variables, while the correlation coefficients of HMW-adn with anthropometric variables in the obesity group were consistently higher than those in the non-obesity group. Moreover, only WHtR was significantly associated with HMW-adn regardless of sex and physique. The results of this study suggested that it is useful to monitor WHtR as a surrogate for HMW-adn among elementary school students, especially obese children.

## Background

Adiponectin is an adipocyte-derived secretory protein that has been very widely studied over the past 15 years [1]. Adiponectin exists as multimers in serum and high-molecular-weight adiponectin (HMW-adn) is particularly considered to be the active form of the protein [2]. A recent population-based study in children reported that HMW-adn is associated with insulin resistance [3]. In addition, recent studies showed that the ratio of HMW-adn to total adiponectin (T-adn) may be as efficient as HMW-adn to evaluate the presence of insulin resistance and metabolic syndrome (MetS) [4], while the ratio of HMW-adn to T-adn (HMW-adn/T-adn) has better predictive power for the prediction of insulin resistance and MetS than plasma T-adn level alone [5]. In fact, measuring HMW-adn was reported to be an efficient method for predicting the progression to MetS compared with measuring T-adn [2]. However, it could be difficult to use HMW-adn for the screening of insulin resistance and MetS in population-based schoolchildren, because blood collection is invasive and is not commonly conducted in the annual health examination for schoolchildren in Japan.

On the other hand, measurement of anthropometric variables including height, weight, percent body fat (%BF), and waist circumference (WC) is non-invasive and easier than the measurement of HMW-adn. Therefore, if some anthropometric variables showed a close correlation with HMW-adn or HMW-adn/T-adn, they could be useful as surrogates for HMW-adn or HMW-adn/T-adn among population-based schoolchildren.

Previous studies have shown the relationship between T-adn and anthropometric variables among population-based children [6,7], while the association between HMW-adn and anthropometric variables have been evaluated among hospital-based children [8,9]. However, studies about the relationship between HMW-adn or HMW-adn/T-adn and anthropometric variables among population-based elementary schoolchildren have been limited, especially in Japan, where blood collection is not usually performed in the annual health examination at elementary schools. Therefore, further evidence for the association between HMW-adn or HMW-adn/T-adn and anthropometric variables is needed among population-based elementary schoolchildren. We hypothesized that the waist-to-height ratio (WHtR) may be the more effective index for predicting HMW-adn or HMW-adn/T-adn level than body mass index (BMI), %BF, and WC.

Accordingly, the aim of the present study was to investigate the relationship of HMW-adn and HMW-adn/T-adn to anthropometric variables among population-based elementary schoolchildren in Japan.

## Methods

The town of Ina, which is located in Saitama Prefecture, Japan, has conducted a unique health-promotion program since 1994, in addition to the annual national health checkups performed in accordance with the School Health Law of Japan. The program consists of a questionnaire survey along with blood and physical examinations for fourth and seventh graders. Several studies of this program have been reported [10-12]. The present study was conducted as part of this program.

### Study subjects

Subjects comprised all fourth-grade schoolchildren (9 or 10 years of age) from Ina during 2005–2008. Informed consent prior to participation in the study was obtained from each subject’s parent or guardian. This study protocol was approved by the two independent institutional review boards at Showa University School of Medicine and Jikei University School of Medicine.

Among 1675 subjects, 13 refused to participate in the program (participation rate: 99.2%) and 8 were excluded because of incomplete data. Thus, data from a total of 1654 subjects (846 boys and 808 girls) were analyzed.

### Biochemical measurement

Blood samples were drawn from the subjects to measure adiponectin isoform values. Adiponectin isoform values were measured using a commercially available enzyme-linked immunosorbent assay kit (Daiichi Pure Chemical Co. Ltd., Tokyo, Japan) [12]. Intraassay coefficients were reported to be 5.3% for T-adn and 3.3% for HMW-adn [13]. The blood collection for study subjects was conducted in the morning after eating breakfast.

### Anthropometric measurements

The height and weight of each subject were measured in the school’s infirmary or in a designated room to protect the subject’s privacy during the procedures. For the anthropometric measurements, the subjects wore light clothing but no shoes or socks. Height was measured to the nearest 0.1 cm using a stadiometer and body weight was measured to the nearest 0.1 kg using a scale. BMI was calculated as weight (kg) divided by height (m) squared. The %BF was measured with a bipedal biometrical impedance analysis device (Model TBF-102, Tanita, Tokyo, Japan) to the nearest 0.1%, over light clothing in a standing position. Subjects were instructed to avoid vigorous activity prior to the measurements. The WC was measured in a standing position at the navel level while another examiner checked verticality from the side. The measurement of WC was performed one time for each subject. The WHtR was calculated as WC divided by height. These measurements were recorded annually from 2005 to 2008.

### Questionnaire survey

The following information was collected using a self-administered questionnaire completed by each child: sex, age, and exercise other than physical education class (daily, sometimes, none). The parent or guardian of each subject was asked to complete the self-administered questionnaire regarding the subject’s birth weight.

### Definition of obesity

Childhood obesity was determined according to the age- and sex-specific cut-off points proposed by the International Obesity Task Force [14].

### Data analysis

The Shapiro-Wilk test was used to test the normality of distribution. To compare various characteristics between subgroups, the Wilcoxon rank-sum test or chi-squared test was used. Spearman’s correlation coefficients between adiponectin levels (HMW-adn, T-adn, and HMW-adn/T-adn) and anthropometric variables (BMI, %BF, WC, and WHtR) were calculated, because adiponectin levels were not normally distributed. The correlation coefficient was applied for each sex. A *P* value of less than 0.05 was considered statistically significant and a value of 0.05 ≤*P* < 0.1 was considered as marginally significant. All statistical analyses were performed using Statistical Analysis System (Version 9.2; SAS Institute Inc., Cary, NC, USA) software.

## Results

The characteristics of the boys and girls studied are shown in Table 1. There was a statistically significant difference between boys and girls in birth weight. Boys were heavier than girls. In addition, BMI, %BF, and WC were higher among boys than among girls. HMW-adn and T-adn in boys were lower than those in girls, although no statistical significance was observed. There was no significant difference between boys and girls in HMW-adn/T-adn. Obesity was more frequently found among boys.

**Table 1:** Baseline characteristics of study subjects

	**Boys**	**Girls**	***P *value**^**a**^
	**(n = 846)**	**(n = 808)**	
Age (years)	9.0 (9.29)	9.0 (9.32)	0.114
Birthweight (g)	3092.0	3028.0	<0.001
Height (cm)	134.9	134.3	0.193
Weight (kg)	30.2	29.4	<0.001
Body mass index (kg/m^2^)	16.6	16.3	<0.001
Percent body fat (%)	18.4	15.8	<0.001
Waist circumference (cm)	57.5	57.3	0.037
Waist-to-height ratio	0.43 (0.44)	0.43 (0.43)	0.066
Proportion of obesity^b^ (%)	3.8	2.0	0.029
HMW-adn (μg/mL)	2.65	2.83	0.496
T-adn (μg/mL)	6.13	6.48	0.249
HMW-adn/T-adn	0.44 (0.433)	0.44 (0.431)	0.856

HMW-adn: High molecular weight adiponectin, T-adn: Total adiponectin, HMW-adn/T-adn: The ratio of HMW-adn to T-adn.

Values are median (mean) or percent.

^a^Wilcoxon rank-sum test or chi-squared test.

^b^Obesity was defined by the criteria of the International Obesity Task Force.

Table 2 shows the correlations of adiponectin levels with anthropometric variables for each sex. High-molecular-weight adiponectin was negatively correlated with BMI, %BF, WC, and WHtR among boys (-0.089 ≤ r ≤ -0.176), while there were also negative correlations between T-adn and each anthropometric variable (-0.097 ≤ r ≤ -0.176). Furthermore, significantly negative correlations were observed between HMW-adn/T-adn and anthropometric variables other than %BF. Among girls, each anthropometric variable was significantly correlated with adiponectin levels (HMW-adn, T-adn, and HMW-adn/T-adn); the range of the correlation coefficients was from -0.111 to -0.188 for HMW-adn, from -0.112 to -0.182 for T-adn, and from -0.099 to -0.168 for HMW-adn/T-adn.

**Table 2:** Correlations of adiponectin levels with anthropometric variables for each sex

	**vs. HMW-adn**		**vs. T-adn**		**vs. HMW-adn/T-adn**	
	**r**^**a**^	***P *value**	**r**^**a**^	***P *value**	**r**^**a**^	***P *value**
Boys (n = 846)						
Body mass index	-0.144	<0.001	-0.139	<0.001	-0.134	<0.001
Percent body fat	-0.089	0.010	-0.097	0.005	-0.059	0.085
Waist circumference	-0.176	<0.001	-0.176	<0.001	-0.149	<0.001
Waist-to-height ratio	-0.160	<0.001	-0.165	<0.001	-0.131	<0.001
Girls (n = 808)						
Body mas index	-0.152	<0.001	-0.138	<0.001	-0.147	<0.001
Percent body fat	-0.126	<0.001	-0.118	<0.001	-0.117	<0.001
Waist circumference	-0.188	<0.001	-0.182	<0.001	-0.168	<0.001
Waist-to-height ratio	-0.111	0.002	-0.112	0.002	-0.099	0.005

HMW-adn: High molecular weight adiponectin, T-adn: Total adiponectin, HMW-adn/T-adn: The ratio of HMW-adn to T-adn.

^a^Values are Spearman’s correlation coefficients.

The comparison of adiponectin levels between the non-obesity and obesity groups is illustrated in Table 3. In boys, HMW-adn, T-adn, and HMW-adn/T-adn levels among the obesity group were significantly lower than those among the non-obesity group. In girls, adiponectin levels were also significantly lower in the obesity group.

**Table 3:** Comparison of adiponectin levels between the non-obesity and the obesity groups by each sex

	**Boys**			**Girls**		
	**Non-obesity**	**Obesity**	***P *value**^**a**^	**Non-obesity**	**Obesity**	***P *value**^**a**^
	**(n = 814)**	**(n = 32)**		**(n = 792)**	**(n = 16)**	
HMW-adn (μg/mL)	2.70	1.99	<0.001	2.88	1.45	<0.001
T-adn (μg/mL)	6.18	4.78	<0.001	6.54	4.45	<0.001
HMW-adn/T-adn	0.44	0.38	<0.001	0.44	0.31	<0.001

HMW-adn: High molecular weight adiponectin, T-adn: Total adiponectin, HMW- adn/T-adn: The ratio of HMW-adn to T-adn.

Values are median.

Obesity was defined by the criteria of the International Obesity Task Force.

^a^Wilcoxon rank-sum test.

The correlation coefficients between adiponectin levels and anthropometric indices by physique (obesity or non-obesity) were calculated in boys (Table 4). Correlation coefficients of adiponectin levels with anthropometric variables in the obesity group were consistently higher than those in the non-obesity group. In addition, only WHtR was significantly correlated with HMW-adn or T-adn regardless of physique; the correlation coefficient with HMW-adn was -0.386 among the obesity group and -0.124 among the non-obesity group, while the correlation coefficient with T-adn was -0.374 among the obesity group and -0.130 among the non-obesity group. When the correlation of HMW-adn/T-adn with each anthropometric variable was evaluated by physique, only WHtR was significantly or marginally significantly correlated with HMW-adn/T-adn.

**Table 4:** Correlation coefficients of adiponectin levels with anthropometric variables by the non-obesity or obesity among boys

	**vs. HMW-adn**		**vs. T-adn**		**vs. HMW-adn/T-adn**	
	**Non-obesity**	**Obesity**	**Non-obesity**	**Obesity**	**Non-obesity**	**Obesity**
	**(n = 814)**	**(n = 32)**	**(n = 814)**	**(n = 32)**	**(n = 814)**	**(n = 32)**
Body mass index	-0.107^a^	-0.212	-0.102^a^	-0.156	-0.104^a^	-0.270
Percent body fat	-0.049	0.064	-0.058^b^	-0.071	-0.024	-0.141
Waist circumference	-0.142^a^	-0.317^b^	-0.142^a^	-0.305^b^	-0.121^a^	-0.225
Waist-to-height raio	-0.124^a^	-0.386^a^	-0.130^a^	-0.374^a^	-0.101^a^	-0.298^b^

HMW-adn: High molecular weight adiponectin, T-adn: Total adiponectin, HMW-adn/T-adn: The ratio of HMW-adn to T-adn.

Obesity was defined by the criteria of the International Obesity Task Force.

Values are Spearman's correlation coefficients.

^a^P < 0.05.

^b^0.05 ≤ P < 0.10.

Correlations of adiponectin levels with anthropometric variables by physique were calculated in girls (Table 5). Correlation coefficients of adiponectin levels were consistently higher in the obesity group than in the non-obesity group. Only WHtR was significantly or marginally significantly associated with adiponectin levels regardless of physique.

**Table 5:** Correlation coefficients of adiponectin levels with anthropometric variables by the non-obesity or obesity among girls

	**vs. HMW-adn**		**vs. T-adn**		**vs. HMW-adn/T-adn**	
	**Non-obesity**	**Obesity**	**Non-obesity**	**Obesity**	**Non-obesity**	**Obesity**
	**(n = 792)**	**(n = 16)**	**(n = 792)**	**(n = 16)**	**(n = 792)**	**(n = 16)**
Body mass index	-0.125^a^	-0.485^b^	-0.111^a^	-0.640^a^	-0.122^a^	-0.353
Percent body fat	-0.098^a^	-0.200	-0.091^a^	-0.281	-0.091^a^	-0.147
Waist circumference	-0.162^a^	-0.381	-0.157^a^	-0.501^a^	-0.144^a^	-0.338
Waist-to-height ratio	-0.081^a^	-0.543^a^	-0.083^a^	-0.589^a^	-0.071^a^	-0.427^b^

HMW-adn: High molecular weight adiponectin, T-adn: Total adiponectin, HMW-adn/T-adn: The ratio of HMW-adn to T-adn.

Obesity was defined by the criteria of the Intenational Obesity Task Force.

Values are Spearman’s correlation coefficients.

^a^P < 0.05.

^b^0.05 ≤ P < 0.10.

## Discussion

In the present study, HMW-adn and HMW-adn/T-adn were negatively correlated with anthropometric variables (BMI, %BF, WC, and WHtR). Furthermore, only WHtR was significantly or marginally significantly correlated with HMW-adn or HMW-adn/T-adn regardless of sex and physique. To the best of our knowledge, this study is the first study regarding the relationship between HMW-adn or HMW-adn/T-adn and anthropometric variables among over 1600 population-based elementary schoolchildren in Japan, where blood collection and the measurement of %BF and WC are not commonly performed in the annual health examination at elementary schools.

In this study, statistically significant differences between boys and girls were found in most anthropometric variables (Table 1). For example, %BF among boys was higher than that among girls in the present study, which was consistent with other study results conducted among Japanese children 9–10 years of age [15,16]. A previous study showed that sexual differences in body composition are present very early in life but emerge most dramatically during puberty [17]; females enter puberty earlier and undergo a more rapid pubertal transition, whereas boys have a substantially longer growth period. Therefore, we analyzed the data separately for each sex to consider sex differences of anthropometric indices and then examined the relationship of each anthropometric variable to adiponectin levels.

As shown in Table 2, anthropometric variables (BMI, %BF, WC, and WHtR) were negatively correlated with HMW-adn and HMW-adn/T-adn. Additionally, HMW-adn and HMW-adn/T-adn were significantly lower in the obesity group than in the non-obesity group regardless of sex (Table 3). Even if the definition of obesity in a recent study was applied to this study, HMW-adn and HMW-adn/T-adn levels in the obese group were still significantly lower than those in the non-obese group for both sexes [18]. A recent study showed that HMW-adn and HMW-adn/T-adn were negatively correlated with BMI among adults [19], while they were reported to be inversely correlated with WC among children [9]. Furthermore, some studies reported that HMW-adn and HMW-adn/T-adn levels in obese children were significantly lower than those in non-obese children [9,20]. Therefore, our study results were consistent with other study results.

In the analysis for each physique, the correlation coefficients of adiponectin levels (HMW-adn, T-adn, and HMW-adn/T-adn) with anthropometric variables differed by physique in each sex (Table 4 and Table 5); the correlation coefficients in the obesity groups were consistently higher than those in the non-obesity groups. The propensity was similar even if stepwise multiple regression analysis (each anthropometric variable, age, and birth weight were included as independent variables) was performed to analyze the relationship between each adiponectin level (each adiponectin level was log-transformed before analysis) and each anthropometric variable. These results suggested that the impact of changing anthropometric variables on adiponectin levels was stronger among the obese students than among the non-obese students. The adiponectin level was reported to be inversely correlated with visceral adipose tissue area [21,22]. Therefore, the amount of increasing or decreasing visceral adipose tissue observed in relation to changing anthropometric variables in the obese students could be larger than that in the non-obese students, resulting in the physique difference seen in the relationship between adiponectin levels and anthropometric variables. Although further study is needed to elucidate the biological mechanism of this physique difference, it could be necessary to consider physique as well as sex when investigating the relationship between adiponectin levels and anthropometric variables.

Moreover, only WHtR was significantly or marginally significantly correlated with adiponectin levels, including HMW-adn, regardless of sex and physique, as shown in Tables 4 and 5. Even if stepwise multiple regression analysis (each anthropometric variable, age, and birth weight were included as independent variables) was performed to analyze the relationship between each adiponectin level (each adiponectin level was log-transformed before analysis) and each anthropometric variable, only WHtR was significantly or marginally significantly correlated with each adiponectin level regardless of physique in girls. Previous studies have shown that WHtR was strongly associated with visceral adipose tissue [23], while adiponectin was inversely associated with visceral adipose tissue [21,22]. Furthermore, WHtR has been reported to be a stronger predictor of visceral adipose tissue than BMI or WC among adults [24,25]. Therefore, WHtR could be the most useful anthropometric variable for predicting adiponectin levels, which suggests that the monitoring of WHtR is an effective tool for the prevention of MetS, especially among obese students, because the correlation coefficients were consistently higher in the obesity groups than in the non-obesity groups. A recent study reported that WHtR is useful to identify overweight children at higher metabolic and cardiovascular risk [26]. In addition, WHtR was reported to be the most significant predictor for total cholesterol, triglyceride, and low-density-lipoprotein cholesterol in Japanese schoolchildren [27], while a past study reported that WHtR is a simple and practical anthropometric index for identifying higher metabolic risks in normal and overweight Japanese men and women [28]. Moreover, Fujita et al. suggested that BMI, WC, and WHtR are highly accurate indicators of excess abdominal fat in Japanese schoolchildren, demonstrating that anthropometric measures are useful indices for school screening and that WHtR is particularly useful because it is not dependent on age or sex and is easy to use [29].

In our study, the majority of the correlations of adiponectin levels with anthropometric variables for each sex were less than |0.200|, which suggested that the correlations might be weak. In contrast, the majority of the correlations of adiponectin levels with WHtR were more than |0.350| in obese boys and more than |0.500| in obese girls. These results showed that it could be helpful to use WHtR as a surrogate for adiponectin levels among the obese, especially among girls.

In the present study, the blood collection from study subjects was conducted in the morning after eating breakfast, which might have affected the data. However, recent studies showed that the level of circulating adiponectin does not change in response to a high-fat meal or 75 g of oral glucose load for serum glucose [30,31]. Accordingly, the study results could not be affected by postprandial status. The second limitation of this study is the lack of information regarding pubertal stage (Tanner’s stage), although it might be technically difficult to obtain the information from more than 1600 population-based children. A previous study reported that the first sign of puberty was testicular growth (≥ 3 ml) in Japanese boys, attained at a mean age of 10.8 years, and breast development (Tanner stage 2) in girls at a mean age of 10.0 years [32]. Because the mean age in the present study participants was 9.3 years old, puberty stage was not likely to have a substantial impact on the results of this study. However, the impact of sex and early pubertal stage on insulin sensitivity and body composition has been reported [33], which might affect our study results. Therefore, to verify our study results, pubertal stage should be considered in future research. Thirdly, our study results might be affected by physical activity. However, there was no statistically significant difference between exercise and adiponectin levels for each sex in the present study (data not shown). Fourth, the number of the subjects in the obesity group was very small compared to that in the non-obesity group. Therefore, it might be difficult to compare the correlation coefficients in the obesity group with those in the non-obesity group, because the results in the obesity group could be more affected by outliers. However, our study results were based on Spearman's rank correlation coefficients and the results were similar even when multiple regression analysis (adiponectin level was log-transformed before analysis) was performed. Accordingly, findings in the present study would be not substantially influenced by outliers. Finally, subjects in our study were from one town in Japan. Therefore, it might be difficult to apply the study results to other races, because ethnic differences in serum adiponectin level have been reported [34-36].

## Conclusions

Adiponectin levels, including HMW-adn and HMW-adn/T-adn, were negatively correlated with BMI, %BF, WC, and WHtR, while correlation coefficients of adiponectin levels with anthropometric variables in the obesity group were consistently higher than those in the non-obesity group for both boys and girls. Moreover, only WHtR was significantly associated with HMW-adn and T-adn regardless of sex and physique. The results of this study suggested that it is useful to monitor WHtR as a surrogate for HMW-adn among elementary school students, especially obese children.

## Abbreviations

HMW-adn: High-molecular-weight adiponectin; T-adn: Total adiponectin; HMW-adn/T-adn: The ratio of high-molecular-weight adiponectin to total adiponectin; %BF: Percent body fat; WC: Waist circumference; WHtR: Waist-to-height ratio; MetS: Metabolic syndrome.

## Competing interests

The authors declare that they have no competing interests.

## Authors’ contributions

HO and TS planned this study. RN and AM contributed to improve the study in a meaningful way. HO drafted this manuscript. TS, RN, and AM performed the data collection. HH supported the data collection. TS supervised the data collection. HO, TO, and AK contributed to the statistical analysis. NT and AK made substantial contributions to the conception of the present study and the revision of the manuscript. All authors read and approved the final manuscript.

## Pre-publication history

The pre-publication history for this paper can be accessed here:

http://www.biomedcentral.com/1471-2431/12/139/prepub
